# Казуистические случаи карциномы околощитовидной железы при верифицированной мутации в гене <i>MEN1</i>

**DOI:** 10.14341/probl13176

**Published:** 2023-02-25

**Authors:** С. В. Пылина, Е. И. Ким, Е. В. Бондаренко, Ю. А. Крупинова, А. К. Еремкина, Н. Г. Мокрышева

**Affiliations:** Национальный медицинский исследовательский центр эндокринологии; Национальный медицинский исследовательский центр эндокринологии; Национальный медицинский исследовательский центр эндокринологии; Национальный медицинский исследовательский центр эндокринологии; Национальный медицинский исследовательский центр эндокринологии; Национальный медицинский исследовательский центр эндокринологии

**Keywords:** первичный гиперпаратиреоз, рак околощитовидных желез, синдром множественной эндокринной неоплазии 1 типа

## Abstract

Рак околощитовидных желез (ОЩЖ), как правило, является спорадическим, однако встречается и при наследственных синдромах. Распространенность карциномы среди пациентов с первичным гиперпаратиреозом составляет около 1% случаев. Сложность диагностики рака ОЩЖ заключается в отсутствии надежных предоперационных предикторов. Клиническая картина чаще всего неспецифична и в целом обусловлена симптомами гиперкальциемии. Данный диагноз рекомендуется устанавливать по результатам морфологического исследования только при наличии истинных признаков инвазии, при этом иммуногистохимическое исследование может быть использовано лишь в качестве вспомогательного метода диагностики. Случаи рака ОЩЖ в рамках синдрома множественных эндокринных неоплазий 1 типа (МЭН-1) крайне редки, поэтому диагностический поиск может быть сопряжен с рядом трудностей. Мы представляем описание двух клинических случаев пациенток с раком ОЩЖ и верифицированными гетерозиготными мутациями в гене MEN1. Описанные нами случаи демонстрируют трудности морфологической диагностики рака ОЩЖ, гетерогенность клинических проявлений при мутации в гене MEN1, а также необходимость своевременного скрининга на наличие других компонентов синдрома МЭН-1 и мутаций в гене MEN1 у родственников первой линии родства.

## Abstract (en)

Parathyroid cancer (PTC) is usually sporadic; however, it could be presented as a component of hereditary syndromes. The prevalence of PTC among patients with primary hyperparathyroidism (PHPT) is about 1% cases. The lack of reliable preoperative predictors significantly complicates the diagnosis of PTC. The clinical course is non-specific and in most cases is determined by severe hypercalcemia. The final diagnosis can only be made on the basis of invasive histopathologic features, while an analysis immunohistochemical (IHC) one can be used only as an additional method. Given the rarity the diagnosis of MEN1-related PTC a challenge. We present two clinical cases of patients with PTC and a verified heterozygous mutation in the MEN1 gene. The described cases demonstrate the complexity of morphological diagnosis for PTC, the heterogeneity of clinical manifestations in patients with the MEN1 mutation, as well as the need for timely screening to identify other components of MEN1 syndrome and mutations of the MEN1 gene among first-line relatives.

## АКТУАЛЬНОСТЬ

Рак околощитовидных желез (ОЩЖ) в структуре первичного гиперпаратиреоза (ПГПТ) составляет в среднем около 1% и характеризуется более тяжелым клиническим течением, большей частотой гиперкальциемических кризов в сравнении с доброкачественными образованиями [1]. Впервые он был описан Sainton и Millot в 1933 г. [2]. Рак ОЩЖ, как правило, носит спорадический характер и редко встречается в рамках наследственных синдромов. В основном он развивается в составе синдрома гиперпаратиреоза с опухолью челюсти (hyperparathyroidism-jaw tumour syndrome, HPT-JT), при котором его частота достигает 15–37,5% [3–5], и семейного изолированного ПГПТ (FIHP) [6–7].

Описаны единичные клинические случаи карциномы ОЩЖ при синдромах множественных эндокринных неоплазий 1 и 2А типов (МЭН-1 и МЭН2А) [8–10]. Известно, что МЭН-1 развивается вследствие потери функции опухолевого супрессора менина, однако злокачественная опухоль ОЩЖ у пациентов с этим заболеванием встречается крайне редко (0,28%) и проявляется клиническими признаками, схожими с таковыми при спорадическом ПГПТ [11]. Мы представляем описание двух клинических случаев рака ОЩЖ у пациенток с гетерозиготными мутациями в гене MEN1, а также краткий обзор литературы по проблеме.

## ОПИСАНИЕ СЛУЧАЯ №1

Пациентка Б., 47 лет.

В феврале 2017 г. в связи с возникновением болевого синдрома, сдавления в области шеи, осиплости голоса и боли в костях обратилась за медицинской помощью по месту жительства, где по итогу комплексного обследования впервые установлен диагноз ПГПТ. Уровень паратиреоидного гормона (ПТГ) на момент манифестации составил 614 нмоль/л (15–68), кальция общего — 3,06 ммоль/л (2,2–2,65). При оценке тиреоидного статуса: уровень тиреотропного гормона (ТТГ) 1,11 кМЕ/мл, свободного тироксина (св. Т4) — 11,8 пмоль/л, кальцитонина — менее 2 пг/мл. По данным УЗИ в нижних отделах правой доли щитовидной железы (ЩЖ) размерами 24×22×59 мм (объем 16 см3) визуализировалось образование размерами 38×25×26 мм, изоэхогенное, неоднородной солидно-кистозной эхоструктуры, васкуляризированное в режиме цветного дуплексного картирования. В левой доле размерами 55×29×80 мм (объем 64 см3) в нижней трети выявлены деформация контура доли и анэхогенное кистозное образование размерами 54×40×61 мм, с четкими контурами, неоднородной эхоструктуры за счет васкуляризации пристеночного солидного участка и наличия множественных септ. Паратрахеально слева позади левой доли железы обнаружено солидное образование с неровными контурами, размерами 30×15×17 мм, средней эхогенности и неоднородной структуры. Лимфатические узлы без особенностей. Тонкоигольная аспирационная биопсия (ТАБ) узлов не проводилась.

На сцинтиграфии с Тс-99м-технетрилом через 2 ч после введения радиофармпрепарата сохранялась его гиперфиксация в проекциях срединного отдела правой и левой долей ЩЖ, со слабой фиксацией под нижним полюсом левой доли ЩЖ, что не позволило достоверно судить о функциональной активности образований. Дополнительно выполнена КТ органов шеи и грудной полости, по результатам которой в корне левого легкого впервые визуализировано образование в S4, размерами 3,2×2,7×3,0 см, по поводу которого было рекомендовано плановое оперативное лечение, от которого пациентка отказалась.

В апреле 2017 г. проведена тиреоидэктомия с паратиреоидэктомией. При интраоперационной ревизии в нижней и средней третях левой доли ЩЖ визуализировалось узловое образование размером до 6 см, с распространением в верхнее средостение. Дорсальнее средней трети левой доли ЩЖ выявлено образование мягкоэластической консистенции размерами 30×20×15 мм. По задней поверхности нижней трети правой доли ЩЖ — железистое образование солидной структуры размером 3 см, а дорсальнее верхнего полюса правой доли — до 8 мм. При гистологическом исследовании в ткани удаленных ОЩЖ обнаружен мультифокальный рост опухолей. Один из узлов с экспансивным ростом, инвазией в капсулу железы и прилежащую мышечную ткань. По данным иммуногистохимического (ИГХ) исследования индекс пролиферативной активности Ki67 составил около 2%, отмечалась положительная экспрессия цитокератина-7, -8 (CK7, СК8), хромогранина А, ввиду чего образование было расценено как карцинома ОЩЖ. Изменения в ткани ЩЖ соответствовали многоузловому зобу с вторичными изменениями.

В послеоперационном периоде отмечались судороги, эпизоды гипокальциемии, вследствие чего назначались препараты кальция и витамина D, способствовавшие стабилизации состояния.

В течение последующих 3 лет пациентку активно ничего не беспокоило. С июля 2020 г. стала отмечать нарастающую общую слабость, кашель. При динамической КТ органов грудной полости выявлена отрицательная динамика роста образования в левом легком до 5,2×3,9×4,8 см. По данным бронхоскопии установлены эпителиальное происхождение и нейроэндокринная дифференцировка опухоли. Индекс пролиферации Ki67 составил 25%. Достоверно дифференцировать нейроэндокринный рак от атипичного карциноида по данным представленного материала не представилось возможным. Также отмечалась положительная экспрессия синаптофизина, регулятора апоптоза Bcl2, хромогранина А, цитокератина и отрицательная — тиреоидного фактора транскрипции 1 (TTF-1).

Учитывая клинико-анамнестические данные, была рекомендована консультация онколога. Проведена КТ органов грудной и брюшной полостей с внутривенным контрастированием, по результатам которой во внутреннем отделе язычковых сегментов левого легкого на уровне верхней легочной вены определялись патологические мягкотканные массы с бугристыми контурами, неравномерно накапливающие контрастный препарат, размерами до 51×47 мм. В брюшной полости и забрюшинном пространстве визуализированы множественные образования: на уровне малой кривизны желудка конгломерат размерами 41×38 мм, по задней стенке селезеночного изгиба толстой кишки образование 38×44 мм, по переднему контуру хвоста поджелудочной железы (ПЖ) до 29 мм, на уровне левого надпочечника 16×26 мм. По переднему контуру головки ПЖ определялось объемное образование 31×34 мм, структура которого была неоднородна за счет наличия жидкостного компонента и кальцинатов. По месту жительства инициировано 3 курса полихимиотерапии этопозидом и карбоплатином AUC 6 (с июля по сентябрь 2020 г.), после завершения которых пациентка самостоятельно обратилась в другое специализированное онкологическое учреждение для получения второго мнения. В рамках данного обращения произведен пересмотр препаратов ОЩЖ от 2017 г., по результатам которого опухоль расценена как аденома ОЩЖ. При оценке биопсийного материала легкого (от июля 2020г.) подтвержден иммунофенотип нейроэндокринной опухоли легкого G2. По данным анализов крови выявлено повышение уровня хромогранина А до 210,4 мкг/л (норма до 100), дофамина — до 47 пг/мл (норма до 44). Онкологом-химиотерапевтом назначен аналог соматостатина длительного действия ланреотид 120 мг п/к 1 раз в 28 дней. Ввиду нейроэндокринного происхождения опухоли в легком рекомендованы проведение позитронно-эмиссионной томографии, совмещенной с компьютерной томографией (ПЭТ/КТ) с GA68 DOTA-TATE и генетическое обследование на синдром МЭН-1.

По данным ПЭТ/КТ всего тела определялась картина 68Ga-DOTA-TATE-позитивных образований: в корне легкого (49×45 мм, Standardized Uptake Value (стандартизированный уровень накопления) SUV=17,39), теле поджелудочной железы (19×13 мм, SUV=6,64), два образования в хвосте ПЖ (11×9 мм, SUV=23,28 и 15×12 мм, SUV 15,22 соответственно) и на уровне малой кривизны желудка (49×36×42, SUV=27,78). 68Ga-DOTA-TATE-негативные образования были обнаружены в головке ПЖ (34×27 мм), в области хвоста ПЖ выявлено не менее трех образований с наибольшим размером в хвосте (до 54×42 мм), а также узловое образование левого надпочечника (29×21 мм).

Проведено секвенирование панели генов «Гиперпаратиреоз», выявлена патогенная мутация в гене MEN1 — 1308G>A р. W436X(NM_130799.2), приводящая к формированию стоп-кодона и преждевременной терминации трансляции белка. В качестве неожиданной находки дополнительно идентифицирована гетерозиготная мутация в гене RET (HG38, chr10:43105062C>T, c.736C>T) с неизвестной клинической значимостью. В связи с выявленным синдромом МЭН-1 проведено генетическое исследование у родной дочери пациентки, по результатам которого определена идентичная мутация в гене MEN1.Среди компонентов синдрома МЭН-1 определялись: ПГПТ, диагноз установлен по месту жительства при случайном обследовании в 2018 г., в том же году проведена лазерная деструкция образования правой нижней ОЩЖ. Из других компонентов синдрома МЭН-1 подтверждена гормонально неактивная микроаденома гипофиза (2021г.).

С ноября 2021 г. пациентка Б. стала регулярно проходить обследование в ОПОЩЖ и НМО ФГБУ «НМИЦ эндокринологии» Минздрава России. При первичной госпитализации основными жалобами были общая слабость, боль в шейном и грудном отделах позвоночника, снижение массы тела на 6 кг за последний год, повышение уровня глюкозы крови до 7 ммоль/л в утренние часы по глюкометру. На тот момент пациентка получала терапию деносумабом 60 мг (инициирована с 2017 г. по месту жительства, показания не установлены, поступление на 3-й месяц после инъекции), ланреотидом 120 мг п/к (11-я инъекция), препаратами цитрата кальция 200–300 мг через день, колекальциферолом 2500 МЕ в день, левотироксином натрия 100 мкг/сут. Кроме того, по назначению гинеколога ввиду ранней хирургической менопаузы с 41 года применялась заместительная гормональная терапия эстроген-гестагенными препаратами. При осмотре визуальные изменения внешности, характерные для акромегалии, гиперкортицизма не выявлены.

Показатели кальциево-фосфорного обмена наиболее соответствовали хроническому послеоперационному гипопаратиреозу. Однако обращали на себя внимание стойкие нормокальциемия и нормокальциурия в отсутствие терапии активными формами витамина D и адекватными дозами кальция, несмотря на гипокальциемический эффект деносумаба (табл. 1). При рентгеноденситометрии отмечен прирост минеральной плотности костей за период 2017–2021 гг. в шейке бедренной кости (-1,3 SD vs. -1 SD), поясничных позвонках (-0,2 SD vs. 0,6 SD) и лучевой кости (-1,8 SD vs. 1,5 SD) по T-критерию. Была рекомендована отмена препарата с последующим решением вопроса о назначении золедроновой кислоты для профилактики синдрома отмены.

В референс-центре патоморфологических исследований ФГБУ «НМИЦ эндокринологии» Минздрава России произведен пересмотр операционного материала ЩЖ и ОЩЖ от 2017 г. (блоки и стекла). В готовых гистологических препаратах определялся рост опухоли солидного, трабекулярного и фолликулярного строения из мелких клеток без четких клеточных границ, со слабо эозинофильной цитоплазмой, округлыми базофильными ядрами без видимых ядрышек. Митотическая активность составила 3 митоза в 10 полях зрения (х400) с атипичной формой деления. В опухоли определялись эмболия тонкостенных кровеносных сосудов, участки фиброза, кровоизлияния, фокусы отложения бурого пигмента и солей кальция, опухоль врастала в собственную соединительную капсулу и прорастала ее. Отмечалась инфильтрация отдельными группами клеток прилежащей жировой клетчатки, без достоверных признаков инвазии опухолью ткани ЩЖ.

По результатам ИГХ-исследования отмечена положительная экспрессия ПТГ, хромогранина A, Ki-67 — 2%, однако, с учетом низкой пролиферативной активности в прилежащем лимфатическом узле, наиболее вероятно индекс Ki-67 нельзя считать достоверным. Таким образом, морфологическая картина соответствовала карциноме ОЩЖ рТ1NхМхPn0LV1 (рис. 1).

В качестве дополнительного метода исследования произведена окраска операционного материала ОЩЖ на парафибромин. Однако полученный результат был сомнительным в связи с низким качеством материала.

В рамках комплексной диагностики впервые проведена МРТ головного мозга с внутривенным контрастированием, по результатам которой выявлена макроаденома гипофиза размерами 5,5×15×10 мм с интраретроселлярным распространением. На 28-й день после инъекции ланреотида зафиксировано повышение уровня инсулиноподобного фактора роста 1 (ИФР-1) до 452,5 нг/мл (табл. 1), а максимальное подавление уровня соматотропного гормона в ходе орального глюкозотолерантного теста составило 1,59 нг/мл. Учитывая полученные лабораторно-инструментальные данные, состояние расценено как активная стадия акромегалии, рекомендованы динамический контроль уровня ИФР-1 и продолжение терапии аналогами соматостатина.

Для динамической оценки множественных нейроэндокринных новообразований (НЭН) выполнена мультиспиральная компьютерная томография (МСКТ) грудной клетки, брюшной полости и забрюшинного пространства. Отмечена отрицательная динамика роста образования в корне левого легкого S4 (поперечный-переднезадний-вертикальный) — 84×38×51 мм по сравнению с данными КТ от июля 2020 г. — 51×47 мм, выявлена инвазия опухолью левой легочной вены (опухолевый тромб 8 мм). В средостении визуализированы многочисленные увеличенные лимфатические узлы. В брюшной полости подтверждены многочисленные гиперваскулярные образования в ПЖ, парапанкреатической клетчатке, двенадцатиперстной кишке. Отмечены увеличение размеров печени (17,6×11,1×16,3 см), объемное образование левого надпочечника и узелковая гиперплазия надпочечников.

Ввиду утери пациенткой биопсийного материала легкого от июля 2020 г., отрицательной динамики роста образования в корне легкого и ретроспективной верификации карциномы рекомендована повторная бронхоскопия легких по месту жительства с проведением ИГХ-исследования и окраской на ПТГ для исключения наличия метастаза карциномы ОЩЖ.

С декабря 2021 г. по решению врача-онколога к терапии ланреотидом присоединены капецитабин 2000 мг/м2 внутрь в 2 приема в 1–14-й дни и темозоломид 150 мг/м2 внутрь в 10–14-й дни.

При последующих госпитализациях в ОПОЩЖ и НМО были выполнены пересмотр стекол и ИГХ-исследование биопсийного материала легкого от апреля 2022 г. Выявлена положительная реакция опухоли на TTF-1 и ПТГ, что морфологически соответствовало высокодифференцированной опухоли легкого, секретирующей ПТГ, и исключало наличие метастаза карциномы ОЩЖ (рис. 2).

На фоне отмены деносумаба впервые после операции на ОЩЖ зафиксированы выраженная гиперкальциемия и гиперкальциурия в сочетании с низким уровнем ПТГ, повышением уровней остеокальцина и С-концевого телопептида коллагена 1-типа (табл. 1). Проведена сцинтиграфия костей, данных за очаговый остеобластный процесс в костях скелета не получено, определялось умеренное повышение метаболизма в позвоночнике и костях таза. С учетом комплекса обследований (в том числе положительной экспрессии ПТГ при ИГХ-исследовании в опухоли легкого), было предположено, что генез гиперкальциемии обусловлен эктопической продукцией ПТГ-подобных пептидов в НЭН легкого. Для коррекции гиперкальциемии и профилактики костных осложнений рекомендованы возобновление инъекций деносумаба в дозе 60 мг п/к, контроль показателей фосфорно-кальциевого обмена в динамике.

На фоне проводимой полихимиотерапии капецитабином и темозоломидом в течение 6 мес наблюдалась стабилизация процесса (без динамики роста множественных НЭН), в связи с чем терапия была продолжена.

Кроме того, при последующем контроле подтверждена частичная резистентность соматотропиномы к аналогам соматостатина (зафиксировано повышение уровня ИФР-1 на 9–10-е сутки после инъекции ланреотида, табл. 1). По данным динамической МРТ через 6 мес значимых изменений размеров образования не было (5×14×8 мм). По согласованию с онкологом-химиотерапевтом было рекомендовано изменение кратности введения ланреотида (120 мг п/к 1 раз в 21 день), а также дополнительное назначение каберголина 0,5 мг 1 раз в неделю.

В связи с выявленными ранее изменениями в показателях кортизола крови и мочи дополнительно проводилось комплексное обследование на предмет гиперкортицизма (табл. 1). С учетом полученных результатов, умеренное повышение кортизола в утренней сыворотке крови и в суточной моче расценено как изменения функционального характера. Учитывая активную стадию акромегалии, не исключался атипически протекающий, периодически манифестирующий АКТГ-независимый гиперкортицизм. Рекомендован динамический контроль.

## ОПИСАНИЕ СЛУЧАЯ №2

Пациентка Н., 37 лет.

Из анамнеза известно, что с 2008 г. наблюдалась эндокринологом по месту жительства по поводу узлового нетоксического зоба, визуально увеличение ЩЖ стала отмечать с 2016 г. По результатам УЗИ от 2016 г. общий объем ЩЖ составил 27,8 см3, в правой доле визуализировалось гипоэхогенное округлое образование с четкими контурами и смешанной васкуляризацией размерами 30×14,1×16,9 мм, в левой доле — образование с гипоэхогенным ободком, размерами 38×23,3×24,9 мм и смешанной васкуляризацией. По данным ТАБ узлов — коллоидный зоб (Bethesda II).

В мае 2021 г. при выполнении УЗИ отмечена отрицательная динамика, объем ЩЖ увеличился до 32,3 см3, преимущественно за счет образования по заднему контуру правой доли ЩЖ, демонстрировавшего неоднородную структуру с гипоэхогенным ободком, размерами 48,7×30,1×41,0 мм, смешанной васкуляризации. По данным лабораторного обследования: кальцитонин менее 1 пг/мл (менее 5), ТТГ 1,75 мкМЕ/мл (0,23–5,33). В связи с появлением жалоб на судороги, ощущение «ползания мурашек по рукам», повышение АД до 140/90 мм рт.ст., сопровождающееся головной болью, быструю утомляемость, усталость, было рекомендовано определение сывороточного уровня кальция, который составил 2,81 ммоль/л (2,2–2,65). По результатам последующих лабораторных анализов верифицирован ПГПТ: кальций общ. 2,91 ммоль/л (2,15–2,5), фосфор 0,52 ммоль/л (0,81–1,45), ПТГ 652 пг/мл (20–74) (табл. 2).

Пациентке была рекомендована госпитализация в специализированный эндокринологический стационар. При обследовании данных за классические осложнения ПГПТ получено не было. При УЗИ подтверждены эхо-признаки образования правой нижней ОЩЖ (гипоэхогенное неоднородное, размерами 40×20 мм с усиленной васкуляризацией), а также узлового образования левой доли (изоэхогенное, размерами 33×41 мм с неоднородными гипоэхогенными зонами, гиперваскуляризованное, TIRADS 4). При повторной ТАБ узла левой доли ЩЖ — Bethesda IV. По результатам сцинтиграфии с ОФЭКТ-КТ определялись признаки образования нижней правой ОЩЖ, узловое образование левой доли ЩЖ, активно накапливающее 99mТс-технетрил.

В связи с абсолютными показаниями выполнены удаление образования правой нижней ОЩЖ, гемитиреоидэктомия слева. В раннем послеоперационном периоде отмечались судороги в верхних и нижних конечностях, спазм мышц брюшного пресса, была назначена терапия альфакальцидолом 1 мкг утром, карбонатом кальция 1000 мг в сутки с положительным эффектом. По результатам планового гистологического исследования операционного материала диагностирована высокодифференцированная опухоль неопределенного злокачественного потенциала левой доли ЩЖ: образование фолликулярного строения диаметром 4 см с участками ядерной атипии, характерной для папиллярного рака, фокусы скопления ксантомных клеток с отложением бурого пигмента, опухоль окружена неравномерной по толщине фиброзной капсулой с участками врастания без достоверных признаков инвазии. Образование ОЩЖ расценено как атипическая аденома pTis: образование из главных клеток с участками фолликулярного строения, очаговым отеком стромы, капсула опухоли визуализируется на малом фрагменте, неоднородная по структуре с признаками врастания без достоверных признаков инвазии. ИГХ-исследование не проводилось.

В сентябре 2021 г. (через 1 мес после операции) госпитализирована в стационар областной больницы по поводу острой гипокальциемии (табл. 2), проводились в/в инфузии глюконата кальция, доза альфакальцидола увеличена до 4 мкг в сутки, карбоната кальция — до 3000 мг в сутки. При выписке уровень кальция общего составил 2,15 ммоль/л.

В октябре 2021 г. впервые госпитализирована в ОПОЩЖ и НМО ФГБУ «НМИЦ эндокринологии» Минздрава России. При обследовании выявлена гиперкальциемия — альбумин-скорректированный кальций 2,78 ммоль/л (2,15–2,55) при сниженном уровне ПТГ до 8,4 пг/мл (15–65) (табл. 2). Проводилась постепенная редукция доз с положительной динамикой как сывороточного кальция (альбумин-скорректированный кальций 2,16–2,26 ммоль/л), так и уровня ПТГ (23,59 пг/мл). При выписке рекомендован прием альфакальцидола 1 мкг в сутки, карбоната кальция 1000 мг в сутки, колекальциферола 2000 МЕ в сутки. Принимая во внимание манифестацию ПГПТ в молодом возрасте, заподозрен синдром МЭН-1. В рамках скрининга на предмет наличия компонентов синдрома определен ИФР-1 — в пределах референса, пролактин не определялся (регулярный менструальный цикл, 2 самостоятельные беременности в анамнезе). При проведении МСКТ брюшной полости с забрюшинным пространством впервые диагностирован правосторонний нефролитиаз (конкременты до 3,5 мм, плотностью около 810 HU), а также образование в хвосте поджелудочной железы (12×13×12 мм с жидкостным компонентом в центре, не накапливающим контрастный препарат, и гиперваскулярным ободком в артериальную фазу сканирования плотностью до 160 HU), подозрительное в отношение НЭН. С учетом размеров выявленного образования и отсутствия признаков гормональной активности, были рекомендованы повторное МСКТ брюшной полости с контрастом через 6–8 мес, оценка уровня хромогранина А в плановом порядке, амбулаторно.

При пересмотре послеоперационного гистологического материала диагноз атипической аденомы был изменен на карциному правой нижней ОЩЖ (pT1NxMx Pn0 LV1): образование из атипичных главных клеток, формирующих солидные поля, на отдельных участках фолликулярные структуры, с участками отека и фиброза стромы, капсула в препаратах определялась на отдельных участках, отмечались участки «внедрения» опухоли в нее, сомнительные признаки сосудистой инвазии, без митотической активности (рис. 3). При ИГХ-исследовании в опухолевых клетках выявлена положительная экспрессия ПТГ, по результатам оценки экспрессии CD34 — сосудистая инвазия опухоли (рис. 3, Б). Индекс пролиферативной активности Ki-67 составил 2%. Также проведено дополнительное ИГХ-окрашивание гистологического материала на парафибромин, по результатам которого экспрессия сохранена (рис. 4).

В дальнейшем на фоне коррекции лечения показатели кальций-фосфорного обмена нормализовались (табл.  2), по результатам УЗИ ЩЖ и ОЩЖ от февраля 2022 г. данных за структурный рецидив заболевания не получено.

С учетом диагноза карциномы ОЩЖ, сохранения экспрессии парафибромина (потеря экспрессии с высокой долей вероятности говорит о мутации в гене CDC73), пациентке было рекомендовано секвенирование панели «Гиперпаратиреоз» (включающей, помимо MEN1, ген CDC73), по результатам которого обнаружена гетерозиготная мутация в 4 экзоне гена MEN1 (c.658T>C, p.Trp220Arg). Вариант не имеет частот в базах данных аллельных вариантов человека, по совокупности сведений замена расценивается как вероятно патогенный вариант (OMIM: 613733). При генетическом обследовании сына (10 лет) и дочери (13 лет) у обоих выявлена идентичная мутация в гене MEN1 (c.658T>C, p.Trp220Arg в 4-м экзоне). Рекомендованы обследование с целью исключения ПГПТ, а также генетическое тестирование других родственников 1-й линии.

**Table:** Таблица 1. Пациентка Б. Кальциево-фосфорный обмен, маркеры костного метаболизма, гормональное обследование

Госпитализации	Ноябрь 2021 г.	Март 2022 г.	Июнь 2022 г.
Показатели кальций-фосфорного обмена
Время, прошедшее после последней инъекции деносумаба	2 мес	7 мес	10 мес
ПТГ интактный (15–65), пг/мл	2,36	6,43	3,42
Кальций общий (2,15–2,55), ммоль/л	2,47	2,61	3,04
Альбумин (35–50), г/л	45	45,1	46
Кальций суточной мочи (2,5–8), ммоль/сут	5,5	5,952	15,863
Кальций скорр., ммоль/л	2,37	2,508	2,92
Фосфор (0,74–1,52), ммоль/л	0,88	1,17	1,1
СКФ, мл/мин/1,73 м2	90	79	80,2
Маркеры костного метаболизма
Остеокальцин (11–43), нг/мл	8,8	-	48,36
С-концевой телопептид коллагена 1-го типа (0,3–0,57), нг/мл	0,13	-	2,02
Щелочная фосфатаза (40–150), Ед/л	47	-	70
Лабораторный скрининг компонентов синдрома и их осложнений
День после инъекции ланреотида п/к 120 мг	28	10	9
ИФР1 (51,0–271,0), нг/мл	401,9	357, 6	452,5
Гликированный гемоглобин, %	6,8	-	-
ГЛК (3,1–6,1), ммоль/л	6,0	6,21	5,92
ГЛК + 120 мин, ммоль/л	6,11	-	-
Максимальное подавление уровня СТГ в ходе ОГТТ 1,59 нг/мл
АКТГ (утро) (7,2–63,3), пг/мл	15,0	25,4	-
Кортизол кровь, утро (171,0–536,0), нмоль/л	639, 5	652,5	-
Кортизол кровь, утро, нмоль/л. НПТ с 1 мг дексаметазона	-	69,3	-
Кортизол мочи (100–379), нмоль/сут	389,4	396,8	-
Кортизол слюны (0,5–9,55), нмоль/л	-	6,6	-
ТТГ (0,250–3,5), мМЕ/л	1,215	2,326	-
Пролактин (69–340), мЕд/л	423,4	-	248,5
Хромогранин (<2)	0,5	-	-
Гастрин (13–115), пг/мл	41,2	-	-
Метанефрин (25–312), мкг/сут	-	-	224,1
Норметанефрин (35–445), мкг/сут	-	-	491,4

**Figure:**
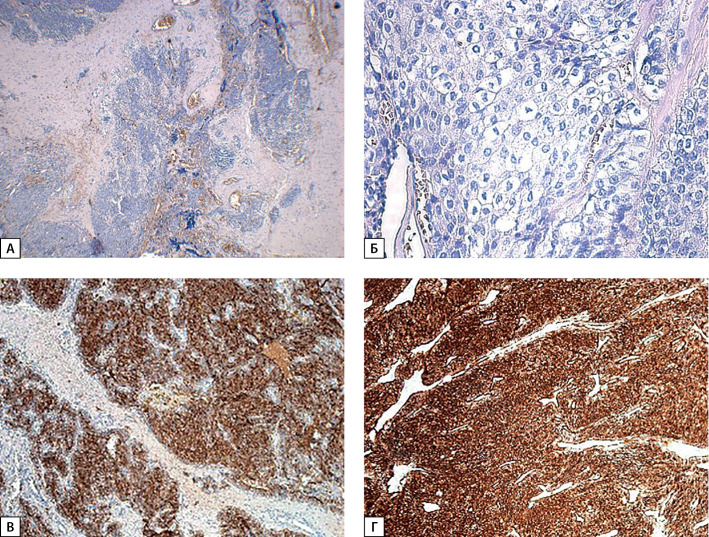
Рисунок 1. Карцинома ОЩЖ (операционный материал от 2017 г.): А — опухоль солидного строения с инвазивным ростом в прилежащие ткани (×40); Б — наличие единичного митоза с атипичной формой деления (×400); В — экспрессия ПТГ; Г — экспрессия хромогранина А.

**Figure:**
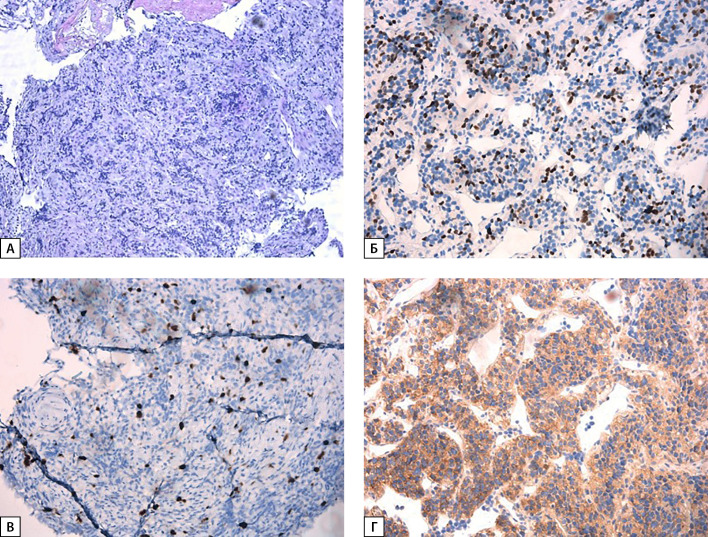
Рисунок 2. Гистологическое исследование бронхоскопии: А — рост опухоли гнездно-трабекулярного строения из небольших относительно мономорфных клеток со скудной светлой эозинофильной цитоплазмой, округлыми и овальными гиперхромными ядрами. Окраска гематоксилин-эозин (×100); Б — положительная реакция с антителом к TTF-1 в части опухолевых клеток. Иммуногистохимическое исследование (×100); В — индекс пролиферативной активности Ki-67 — 10%. Иммуногистохимическое исследование (×100); Г — положительная экспрессия ПТГ в опухолевых клетках. Иммуногистохимическое исследование (×100).

**Table:** Таблица 2. Пациент Н. Динамика лабораторных показателей

Дата/параметр	Са общ., ммоль/л	Фосфор, ммоль/л(0,81–1,45)	Ca в суточной моче, ммоль/сут	ПТГ,пг/мл	ТТГ, мМЕ/л	СКФ (по CKD-EPI), мл/мин/1,73 м2	25(ОН) витамин Dнг/мл
05.2021	2,81(2,2–2,65)	0,37	-	-	-	-	-
06.2021	2.91(2,15–2,5)	0.53	-	652(20–74)	1,75	116	16,82
07.2021	3,06(2,2–2,65)	0,49	9,69(2,5–6,2)	745(16–60)	0,64	-	-
08.2021, после операции	2,01(2,2–2,65)	0,61	-	-	0,1	-	-
09.2021	1,86(2,20–2,65)	-	-	-	-	-	-
10.2021	2,81(2,15–2,55)	1,41	12,6	8,4(15–65)	2,12	89	-
10.2021 (на фоне коррекции лечения)	2,18(2,15–2,55)	-	-	23,59(15–65)	-	-	-
02.2022	2,26	1,09	-	36,58(15–65)	-	114	-

**Figure:**
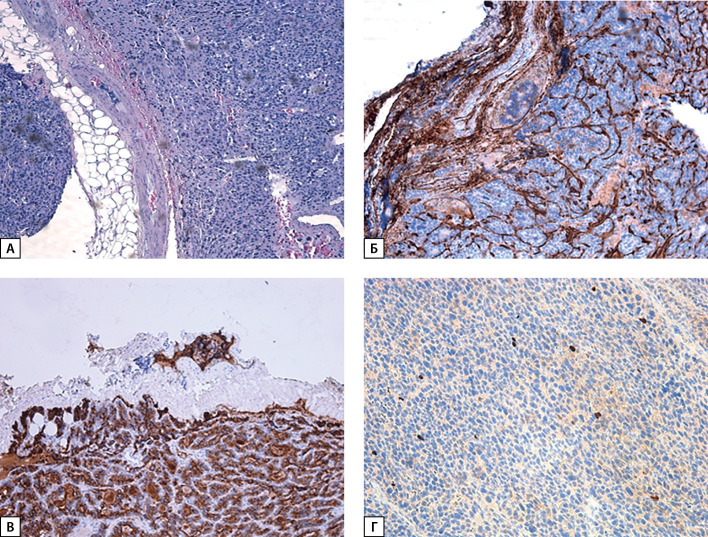
Рисунок 3. А — карцинома ОЩЖ, окраска гематоксилин-эозином (×100); Б — экспрессия CD34, сосудистая инвазия (×100); В — экспрессия ПТГ в опухоли за пределами капсулы; Г — экспрессия Ki-67 2%.

**Figure:**
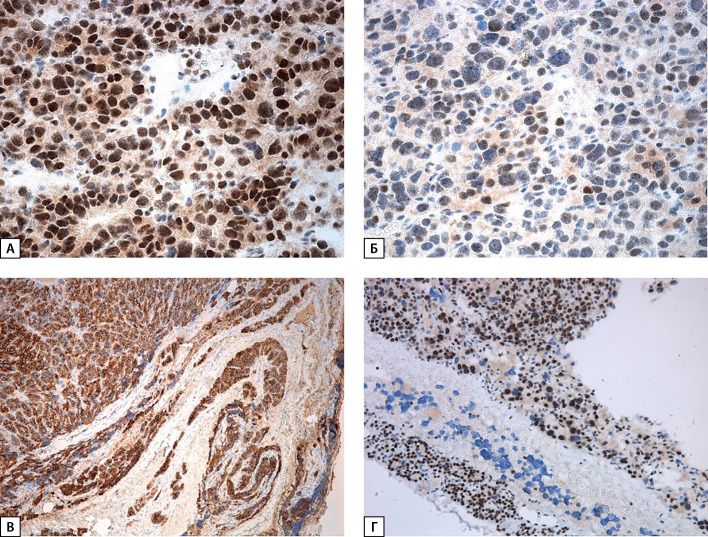
Рисунок 4. Окраска на парафибромин: А, Б — участки с позитивным окрашиванием ядер разной интенсивности и поля цитоплазматической реакции, ×400; В — выраженная ядерная экспрессия в окружающей опухоль паратиреоидной ткани, ×100.

**Table:** Таблица 3. Клинические случаи рака ОЩЖ в составе МЭН-1

№	Автор, год	Пол	Возраст на момент постановки ПГПТ	ПТГ, пг/мл	Кальций, ммоль/л	Количество измененных ОЩЖ	Морфологическая характеристика опухолей ОЩЖ	Метастазы	Другиекомпоненты МЭН	Генетическая мутация	Семейныйанамнез
1	С.Wun Wu, 1992 [13]	М	52	154,3	4,1	1	Рак	Местный рецидив + метастазы в органы грудной клетки	Аденома гипофиза	-	Не отягощен
2	M. Sato, 2000 [14]	Ж	51	Нет данных	2,7	4	1 — рак3 — аденома	Нет	Нет данных	842delCin exon 4	Не отягощен
3	S. Dionisi, 2002 [15]	М	35	707	3,4	1	Рак	Метастазы в средостение	Синдром Золлингера–Эллисона; липома	-	Не отягощен
4	A. Agha, 2007 [16]	Ж	69	342,7	3,9	1	Рак	Рецидив (эктопия ОЩЖ в средостение)	Пролактинома; гормонально-неактивная нейроэндокринная опухоль поджелудочной железы	-	Не отягощен
5	A. Agha, 2007 [16]	М	32	254,5	3,7	1	Рак	Нет	Гастринома; инсулинома гиперплазия надпочечников; синдром Иценко–Кушинга	-	У матери — ПГПТ;у дочери — гипогликемии
6	R. Shih, 2009 [17]	Ж	53	1354	3,4	1	Рак	-	Аденома гипофиза; гастринома; инциденталома надпочечников	c.1406_13dup8	Не отягощен
7	S.Kalavalapalli, 2010 [18]	Ж	44	79,1	2,7	4	-	Метастазы в легкие	ИПФР-1 аденома гипофиза; гормонально-неактивная нейроэндокринная опухоль поджелудочной железы	-	Не отягощен
8	C. del Pozo, 2011 [19]	М	50	204	3,1	1	Рак	Рецидив	Гастринома (злокачественная); гормонально-неактивная аденома гипофиза	W183Cin exon 3	У дочери — мутация c.549G>T
9	L. Juodele, 2011 [20]	Ж	39	311,8	3,3	2	Рак	Рецидив	Микропролактинома; инсулинома (злокачественная); липома; инциденталома надпочечников	c.129insA-codon43in exon 2	У отца — гастринома (причина смерти); у сына — мутация c.129_130insA
10	K. Lee, 2013 [21]	Ж	59	248,2	3,2	1	Рак	Нет	Микроаденомы гипофиза (2); инциденталома надпочечников	-	Нет данных
11	N. Singh Ospina, 2014 [22]	М	62	127,3	3,1	4	1 — рак3 — аденома	Рецидив	Гастринома; рак легких; инциденталома надпочечников	-	Отягощен по МЭН1
12	I. Christakis, 2016 [23]	М	64	42	2,6	4	1 — рак3 — гиперплазия	Нет	Гормонально-активная опухоль поджелудочной железы; рак легких	c.703G > A(p.E235K)	Нет данных
13	I. Christakis, 2016 [23]	М	56	673,1	3,4	4	1 — рак3 — гиперплазия	Нет	Гормонально-активная опухоль поджелудочной железы; инциденталома надпочечников; аденома гипофиза	c.1378C > T(p.R460X)	Нет данных
14	Y. Omi, 2017 [24]	М	40	522	2,74	4	2 — рак2 — гиперплазия	Нет	Гормонально-неактивная аденома гипофиза; опухоль поджелудочной железы	-	У матери — МЭН1 (ПГПТ, пролактинома, гормонально-неактивная опухоль поджелудочной железы; рак легких)
15	L. Cinque, 2017 [25]	М	61	Нет данных	Нет данных	2	1 — рак1 — нет данных	Нет	Инциденталома надпочечников; опухоль поджелудочной железы	c.1252G > A; p.D418 Nin exon 9	
16	L. Cinque, 2018 [25]	М	55	286	1,48	1	Рак	Нет	Инциденталома надпочечников	c.1252G>A; p.D418 Nin exon 9	
17	G. Di Meo, 2018 [26]	М	62	391,7	2,92	1	Рак	Нет	Нет данных	-	У брата ПГПТ, мутация (c.1252 G > A).

## ОБСУЖДЕНИЕ

Поражение ОЩЖ в рамках синдрома МЭН-1 наиболее часто обусловлено гиперплазией ОЩЖ, реже встречаются множественные аденомы. Характер роста гиперплазированных клеток при МЭН-1-индуцированной трансформации ОЩЖ может быть диффузным, узловым или диффузно-узловым [12], что в ряде случаев затрудняет дифференциальную диагностику между типичной аденомой и диффузно-узловой гиперплазией с наличием одного доминантного узла. При этом случаи злокачественного поражения и/или возникновения атипических аденом ОЩЖ при МЭН-1 казуистически редки: на сегодняшний день в литературе описано всего 17 случаев рака ОЩЖ в рамках данного синдрома [13–26] (табл. 3).

Диагноз карциномы ОЩЖ представляет собой серьезный диагностический вызов для широкого круга специалистов. Ввиду отсутствия достоверных предоперационных маркеров заболевания диагноз устанавливается на основании результатов морфологического исследования. Первоначально для диагностики рака ОЩЖ использовались критерии, представленные Schantz и Castleman еще в 1973 г. На основании оценки 70 карцином ОЩЖ ими были предложены капсулярная или сосудистая инвазия, широкие фиброзные тяжи, наличие митозов в паренхиматозной ткани [27]. Однако многие из этих признаков (некрозы, широкие фиброзные тяжи, сращение с соседними структурами без инвазии в них, солидный или трабекулярный тип строения, ядерная атипия) могут наблюдаться и при атипических аденомах ОЩЖ [28]. К тому же широкие фиброзные тяжи могут быть артефактами после ТАБ [29], появляться во время подготовки гистологического материала, а также нередко встречаться у пациентов с синдромом МЭН-1 и МЭН-2 [30]. Поэтому на сегодняшний день в соответствии с критериями, предложенными Всемирной организацией здравоохранения (ВОЗ), 2017 [31], диагноз карциномы ОЩЖ устанавливается только при наличии достоверных признаков инвазивного роста в прилежащие структуры, доказанной сосудистой инвазии и/или документированных метастазов [32]. Тем не менее стоит отметить, что не всегда при гистологическом исследовании удается оценить состояние капсулы на всем протяжении, а признаки сосудистой инвазии могут быть не столь очевидны.

Ранее в исследованиях было показано, что потеря ядерной экспрессии парафибромина имеет специфичность 96% и чувствительность 99% в качестве маркера карциномы ОЩЖ [33]. Белок парафибромин кодирует ген CDC73, ответственный за развитие синдрома HPT-JT и рака ОЩЖ, предполагается, что после биаллельной инактивации этого гена ингибиторный эффект парафибромина на активность циклина D1 теряется, что приводит к неопластической трансформации в ткани ОЩЖ [34]. Однако присутствие экспрессии парафибромина не позволяет полностью исключить возможность мутации в гене CDC73 по причине инактивации в виде точечных мутаций, что может приводить к сохранению экспрессии парафибромина [35]. Ввиду того, что карцинома ОЩЖ встречается редко, но при наличии мутации HPT-JT в 15% случаев [36], генетический скрининг на мутацию в гене CDC73 рекомендуется всем пациентам. Поиск специфических ИГХ-маркеров, подобных парафибромину, способных повысить специфичность гистологического исследования и помочь в дифференциальной диагностике между доброкачественными и злокачественными образованиями ОЩЖ, продолжается. Среди наиболее известных можно выделить Ki-67, галектин-3, PGP9.5, BRCA2 [37], Bcl-2a, Rb [38], p27, PRAD1, циклин D1, COX-1/2, Gst-π и белки сигнального пути sonic hedgehog [39], PAX8, GATA-3, CGA, MyoD1, HMB 45, TFE3, EMA [40], однако их прогностическая ценность остается предметом дискуссий.

В представленных нами клинических случаях постановка диагноза карциномы ОЩЖ основана прежде всего на достоверных признаках инвазивного роста. В первом случае отмечено прорастание опухоли за пределы собственной капсулы, во втором благодаря ИГХ-исследованию и оценке экспрессии CD34 выявлена сосудистая инвазия. С целью уточнения этиологии карциномы ОЩЖ, в качестве дополнительного метода диагностики, проведена оценка экспрессии парафибромина. В первом клиническом случае оценить экспрессию парафибромина не удалось вследствие отсутствия окрашивания внутреннего положительного контроля (эндотелий сосудов) при положительном внешнем контроле, что предположительно свидетельствовало о низком качестве послеоперационного гистологического материала и исключало технические ошибки в протоколе исследования. Во втором случае экспрессия парафибромина была сохранена, что с высокой вероятностью свидетельствовало об отсутствии мутации CDC73.

В обоих клинических случаях наблюдалась прямая зависимость пенетрантности компонентов синдрома МЭН-1 от возраста пациентов, что подтверждается Schaaf L. и соавт. [41]. Поскольку каждое новое проявление синдрома МЭН-1 увеличивает частоту осложнений и снижает качество жизни пациентов, в рамках своевременной диагностики данного заболевания применяется программа генетического скрининга, предложенная Thakker R. и соавт. [42]. Обследование на предмет мутаций в гене MEN1 может быть рекомендовано как пациентам при наличии минимум двух НЭН (клинический случай №1), так и пациентам молодого возраста, у которых выявлен только ПГПТ (клинический случай №2). Поскольку синдром МЭН-1 является аутосомно-доминантным заболеванием с вероятностью передачи дефектного гена в 50% случаев, генетический скрининг рекомендовано проводить среди родственников пробанда, относящихся к первой линии родства. Однако при верифицированной карциноме ОЩЖ проведения секвенирования только в пределах гена MEN1 недостаточно для исключения других наследственных заболеваний, сопровождающихся развитием ПГПТ. В подобной ситуации предпочтительным будет исследование панели генов, ассоциированных с ПГПТ. Проведение секвенирования панели генов «Гиперпаратиреоз» (клинический случай №1) позволило выявить мутации сразу в двух генах — MEN1 и RET. Клиническая картина заболевания в представленном нами случае соответствовала «классическому фенотипу» МЭН-1. И хотя ПГПТ может развиваться при мутациях в гене RET, к преобладающим компонентам синдрома МЭН-2 все же относят медуллярный рак ЩЖ и феохромоцитому, которых у пациентки Б. диагностировано не было. По данным литературы, сочетание данных мутаций может давать вариабельную симптоматику [43][44]. Однако, по мнению Frank-Raue K. и соавт. [45], при установлении мутации с неясной клинической значимостью, как в случае пациентки Б., клинические проявления МЭН-2 могут полностью отсутствовать.

Несмотря на то что на сегодняшний день не доказана взаимосвязь между типом мутации в гене MEN1 и прогнозом течения заболевания, в семьях с синдромом МЭН-1 риск смертности ниже по сравнению с лицами со спорадической формой МЭН-1 [46]. Это свидетельствует о необходимости раннего генетического скрининга и прицельного диспансерного наблюдения в специализированных учреждениях, что позволит улучшить прогноз пациентов.

## ЗАКЛЮЧЕНИЕ

В большинстве случаев поражение ОЩЖ при МЭН-1 представлено гиперплазией, но не стоит забывать о возможности развития рака в качестве единственного или одного из компонентов синдрома. Диагностика рака ОЩЖ основана на выявлении достоверных признаков инвазивного роста. Тем не менее специфичность морфологического исследования для карциномы ОЩЖ может существенно варьировать, что в сомнительных случаях требует использования вспомогательных методов диагностики, в том числе ИГХ-исследования. Генетический скрининг с использованием панели генов, ассоциированных с ПГПТ, способствует ранней постановке диагноза, проведению комплексной диагностики и своевременному лечению.

## ДОПОЛНИТЕЛЬНАЯ ИНФОРМАЦИЯ

Источники финансирования. Работа выполнена в рамках государственного задания «Оптимизация Российского электронного реестра пациентов с первичным гиперпаратиреозом», регистрационный номер 121030100032-7.

Конфликт интересов. Авторы декларируют отсутствие явных или потенциальных конфликтов интересов, связанных с публикацией настоящей статьи.

Участие авторов. Пылина С.В., Ким Е.И. — написание основного текста статьи, анализ литературных данных, Бондаренко Е.В. — получение и интерпретация результатов, проведение ИГХ-исследований, описание препаратов; Крупинова Ю.А. — получение и интерпретация результатов; написание рукописи; Еремкина А.К., Мокрышева Н.Г. — концепция и дизайн исследования, внесение в рукопись существенныхправок. Все авторы одобрили финальную версию статьи перед публикацией, выразили согласие нести ответственность за все аспекты работы, подразумевающую надлежащее изучение и решение вопросов, связанных с точностью или добросовестностью любой части работы/

Согласие пациента. Пациенты добровольно подписали информированное согласие на публикацию персональной медицинской информации в обезличенной форме для публикации в журнале «Проблемы эндокринологии».
